# Evidence for a Cystic Fibrosis Enteropathy

**DOI:** 10.1371/journal.pone.0138062

**Published:** 2015-10-20

**Authors:** Marlou P. M. Adriaanse, Linda J. T. M. van der Sande, Anita M. van den Neucker, Paul P. C. A. Menheere, Edward Dompeling, Wim A. Buurman, Anita C. E. Vreugdenhil

**Affiliations:** 1 Department of Paediatric Gastroenterology & Nutrition and Toxicology Research Institute Maastricht (NUTRIM), Maastricht University Medical Centre, Maastricht, the Netherlands; 2 Department of General Surgery, Maastricht University Medical Centre, Maastricht, the Netherlands; 3 Department of Paediatric Pulmonology, School for Public Health and Primary Care (CAPHRI), Maastricht University Medical Centre, Maastricht, the Netherlands; 4 Department of Immunodiagnostics, Central Diagnostic Laboratory, Maastricht University Medical Centre, Maastricht, the Netherlands; The Hospital for Sick Children and The University of Toronto, CANADA

## Abstract

**Background:**

Previous studies have suggested the existence of enteropathy in cystic fibrosis (CF), which may contribute to intestinal function impairment, a poor nutritional status and decline in lung function. This study evaluated enterocyte damage and intestinal inflammation in CF and studied its associations with nutritional status, CF-related morbidities such as impaired lung function and diabetes, and medication use.

**Methods:**

Sixty-eight CF patients and 107 controls were studied. Levels of serum intestinal-fatty acid binding protein (I-FABP), a specific marker for enterocyte damage, were retrospectively determined. The faecal intestinal inflammation marker calprotectin was prospectively studied. Nutritional status, lung function (FEV1), exocrine pancreatic insufficiency (EPI), CF-related diabetes (CFRD) and use of proton pump inhibitors (PPI) were obtained from the medical charts.

**Results:**

Serum I-FABP levels were elevated in CF patients as compared with controls (p<0.001), and correlated negatively with FEV1 predicted value in children (r-.734, p<0.05). Faecal calprotectin level was elevated in 93% of CF patients, and correlated negatively with FEV1 predicted value in adults (r-.484, p<0.05). No correlation was found between calprotectin levels in faeces and sputum. Faecal calprotectin level was significantly associated with the presence of CFRD, EPI, and PPI use.

**Conclusion:**

This study demonstrated enterocyte damage and intestinal inflammation in CF patients, and provides evidence for an inverse correlation between enteropathy and lung function. The presented associations of enteropathy with important CF-related morbidities further emphasize the clinical relevance.

## Introduction

Cystic fibrosis (CF) is a complex multisystem disease caused by mutations in the CF transmembrane conductance regulator (CFTR) gene, leading to dehydrated luminal secretions and impaired secretion clearance, affecting mainly the respiratory and gastrointestinal tract. The contribution of intestinal involvement in CF to the disease progress and development of complications is largely unknown. It is challenging to achieve and maintain an optimal nutritional status despite nutritional interventions and treatment of exocrine pancreatic insufficiency (EPI) [1, 2, 3]. A compromised gut, with inflammation and enterocyte damage, both associated with malabsorption, may contribute to a poor nutritional status in CF patients [4, 5]. Poor nutritional status results not only in impaired growth but also affects lung function and survival [6, 7]. Furthermore, intestinal damage and inflammation, with consequent loss of barrier function [8], might potentially also adversely affect lung function by translocation of bacteria and their toxins, further aggravating lung inflammation and worse clinical outcome [9, 10].

Evidence for intestinal inflammation in CF has been found in both human and animal studies [5, 11, 12, 13, 14, 15, 16]. Raised levels of cytokines (TNF-α), interleukins (IL-1β, IL-8), immunoglobulins (IgM, IgG), neutrophil elastase and calprotectin were demonstrated in faeces and whole gut lavage of CF patients [5, 11, 12, 13, 16, 17]. Also increased mononuclear cell infiltration of the lamina propria has been shown in duodenal biopsies [15]. Furthermore, intestinal inflammation with concomitant activation of the innate immune system was found in a CF mouse model [14]. Different causes have been suggested for intestinal inflammation in CF patients, such as the CFTR mutation itself leading to an altered innate immunity and a consequent pro-inflammatory state [14, 15, 18]. In addition, EPI potentially resulting in altered intestinal microbiota [19], bacterial overgrowth [20] and swallowed sputum containing pro-inflammatory mediators [11], could contribute to intestinal inflammation in CF.

Also evidence for structural intestinal alterations and damage in the CF intestine has been reported. Mucosal lesions consisting of edema, erythema and erosions were found in both exocrine pancreatic sufficient and insufficient CF patients [5]. Interestingly, ultrastructural lesions are present in those parts of the intestine where molecular studies have described the highest CFTR expression [21]. Additionally, the increased acidity resulting from diminished bicarbonate secretion by the pancreas [22] and the abnormal mucus overlying the intestinal mucosa [23] may affect the intestinal mucosal integrity in CF [8, 24].

This study aimed to evaluate enterocyte damage and intestinal inflammation in CF. Serum intestinal fatty acid binding protein (I-FABP) is used to asses enterocyte damage. I-FABP is a small cytosolic protein exclusively present in the enterocytes of the intestine, with a maximal expression in the jejunum, while the expression in the colon is low [25, 26, 27, 28]. I-FABP is predominantly present at the upper part of the villi, and upon small intestinal damage the protein is released into the systemic circulation. Faecal calprotectin, a marker for neutrophil activation or degradation [29], is used to evaluate intestinal inflammation. Correlations between these quantitative enteropathy measures and disease characteristics of CF were assessed to investigate whether control of intestinal alterations represents a potential therapeutic target for improvement of nutritional status and preservation of lung function.

## Materials and Methods

### Study subjects

All CF patients treated at the Maastricht University Medical Centre between 2004 and 2011 were considered for inclusion. CF diagnosis was based on a typical clinical picture with identification of two CF-disease causing mutations and an abnormal sweat test. Patients with celiac disease and/or inflammatory bowel disease were excluded, since these diseases are associated with intestinal damage and inflammation.

Faecal samples were prospectively collected to determine faecal calprotectin levels. To evaluate whether calprotectin found in faeces in CF patients origins from the lungs, sputum samples were collected on the same day as faeces collection. Per patient, the most recent blood sample for I-FABP analysis was retrospectively selected. Data from CF patients were obtained from the medical charts. Malnutrition was defined as a body mass index (BMI) < 18.5 kg/m^2^ in adults [30] and children (<18 years of age) had to meet one of the following criteria; BMI Z-score <10^th^ percentile [31], weight-for height Z-score <-2 and/or height Z-score <-2. Since it can be hypothesized that a low pH due to reduced bicarbonate secretion might affect the intestine, the presence of EPI was reported. EPI was determined by a low coefficient of fat absorption (<90%), low faecal elastase (<200 μg/g) and/or low faecal chymotrypsin (<6.6 U/g) [32]. Furthermore, CF-related diabetes (CFRD), confirmed by an oral glucose tolerance test [33], was recorded, as both type I and type II diabetes have been associated with intestinal disturbances [34, 35]. *Pseudomonas aeruginosa* (PA) colonization, based on repeated sputum cultures, was registered. C-reactive protein (CRP) level was documented. Also medication use might affect the intestine. In this context, the use of pancreatic enzyme replacement therapy (PERT) and its lipase dose, and proton pump inhibitors (PPI) use were reported.

The control group for serum I-FABP levels consisted of adults and children that had presented with gastrointestinal complaints or failure to thrive at the gastroenterology department or paediatric department in whom celiac disease and inflammatory bowel disease were excluded.

The study protocol was approved by the Medical Ethics Committee of the Academic Hospital Maastricht and Maastricht University, and the study was performed according to their guidelines. Participants provided their written informed consent to participate in this study. On behalf of the children enrolled in this study, written informed consent was obtained from their parents, and children did co-consent if aged 12–17 years.

### Measurement of I-FABP, calprotectin and lung function

Serum from celiac disease screening in CF patients and controls was stored at -20°C until batch analysis. From 56 CF patients, fulfilling the abovementioned criteria, serum was available. Serum I-FABP levels were determined using a highly specific in-house enzyme-linked immunosorbent assay (ELISA) that selectively detects human I-FABP without cross-reaction with other FABP types (standard: 12.5–800 pg/ml). Storage time of serum samples did not influence levels of I-FABP.

Faeces samples were collected at home and stored at room temperature until analysis. Faecal calprotectin could be measured in 43 CF patients. Missing values of faecal calprotectin were caused by patients unwilling to participate for this aspect, or due to failure of the faeces collection or storage. To evaluate whether calprotectin found in faeces origins from the lungs, sputum samples were collected on the same day as faeces collection. Sputum was spontaneously induced and stored at -20°C until analysis. Calprotectin levels were determined using a sensitive and specific commercial available enzyme-linked immunoassay (EliA calprotectin assay, Phadia, Sweden). The upper normal limit is <50 microgram/gram (μg/g) according to the manufacturer.

Dynamic spirometry was performed using the Flowscreen^®^ (Viasys, Hoeckberg, Germany), according to the standards of the American Thoracic Society / European Respiratory Society. Highest values of three correctly performed manoeuvres were used for analysis. Recorded parameters were forced expiratory volume in 1 second (FEV1), forced vital capacity (FVC), both expressed as a percentage of the predicted normal value, and FEV1/VC. Lung function tests were not available in the control group.

### Statistical analysis

Statistical analyses were performed using SPSS 20.0 (SPSS Inc, Chicago, USA). Normally distributed variables were analysed using t-tests (mean with standard deviation (SD)) and Pearson correlation (r_p_), while not-normally distributed date were analysed using Mann-Whitney U-tests (median with range) and Spearman correlation (r_s_). Linear regression was used to model the relationship between a dependent variable and (multiple) independent variables (B, standard error (SE), p-value). All tests were two-tailed and a p-value <0.05 was considered statistically significant.

## Results

### Patient characteristics

Sixty-eight CF patients were eligible for inclusion (Table 1): serum I-FABP levels were analysed in 56 CF patients (I-FABP group, S1 File) whereas faecal calprotectin levels were available in 43 CF patients (calprotectin group, S2 File). Eight young CF children were unable to perform the lung function tests. Hundred and seven subjects were included as controls.

**Table 1:** Characteristics of cystic fibrosis patients and control subjects.

		Cystic fibrosis patients		Control group
		I-FABP group	Calprotectin group	
		n = 56	n = 43	n = 107
		n (%) or median (range)	n (%) or median (range)	n (%) or median (range)
**Sex**	Male	29 (51.8%)	26 (60.5%)*	39 (36.4%)
	Female	27 (48.2%)	17 (39.5%)	68 (63.6%)
**Age, group**	Adults	35 (62.5%)	19 (44.2%)	46 (43.0%)
	Children	21 (37.5%)	24 (55.8%)	61 (57.0%)
**Age, years**		19.6 (0.7–39.1)	16.0 (0.7–46.3)	16.1 (0.5–87.5)
**Delta F508 homozygous**	Yes	31 (55.4%)	27 (62.7%)	
	No	19 (33.9%)	13 (30.2%)	
	unknown	6 (10.7%)	3 (7.0%)	
**Forced expiratory volume in 1 second (FEV1, % predicted)**		58.4 (20.3–106.3)	61.6 (17.5–118.3)	
**Forced vital capacity (FVC % predicted)**		79.4 (35.9–113.5)	79.0 (30–129.9)	
**FEV1/VC (% predicted)**		81.5 (38.8–104.3)	83.3 (35.6–107.9)	
**Body mass index (BMI) (kg/m2)**	Adults	20.5 (15.4–29.1)	20.0 (15.3–29.1)	22.8 (12.5–40.2)
**BMI Z-score**	Children	-.63 (-4.2–3.7)	-.4 (-2.4–3.1)	-.05 (-3.5–3.7)
**Height Z-score**	Children	-1.7 (-3.7–0.26)#	-1.7 (-3.6–0.56)**	-.4 (-3.5–1.8)
**Weight-for-height Z-score**		-.29 (-4.1–4.0)	-.22 (-2.5–3.6)	.3 (-3.1–3.4)
**Malnutrition**		12 (21.4%)	13 (30.2%)	17 (15.9%)
**C-reactive protein (CRP) (mg/l)**		8.9 (0–91.0)	9.0 (1.5–64.0)	
**CF-related diabetes**		13 (23.2%)	9 (20.9%)	
**Exocrine pancreatic insufficiency**		46 (82.1%)	38 (88.4%)	
**Pancreas enzyme replacement therapy (PERT)**		46 (82.1%)	38 (88.4%)	
**Pseudomonas Aerugionosa colonization**		20 (35.7%)	14 (32.6%)	
**Proton pump inhibitor use**		30 (53.6%)	28 (65.1%)	
**Lipase use; Use per kg (mg)**			37 (80.4%); 4820 (1908–18519)	

Abbreviations: I-FABP: intestinal fatty acid binding protein, n: number, FEV1: forced expiratory volume, FVC: forced vital capacity, VC: vital capacity, kg: kilogram, mg: milligram

^**#**^ significant difference between I-FABP group and control group, p < 0.001

* significant difference between the calprotectin group and the control group, p < 0.05

** significant difference between calprotectin group and control group, p < 0.001

### Associations with serum I-FABP

**Fig 1:**
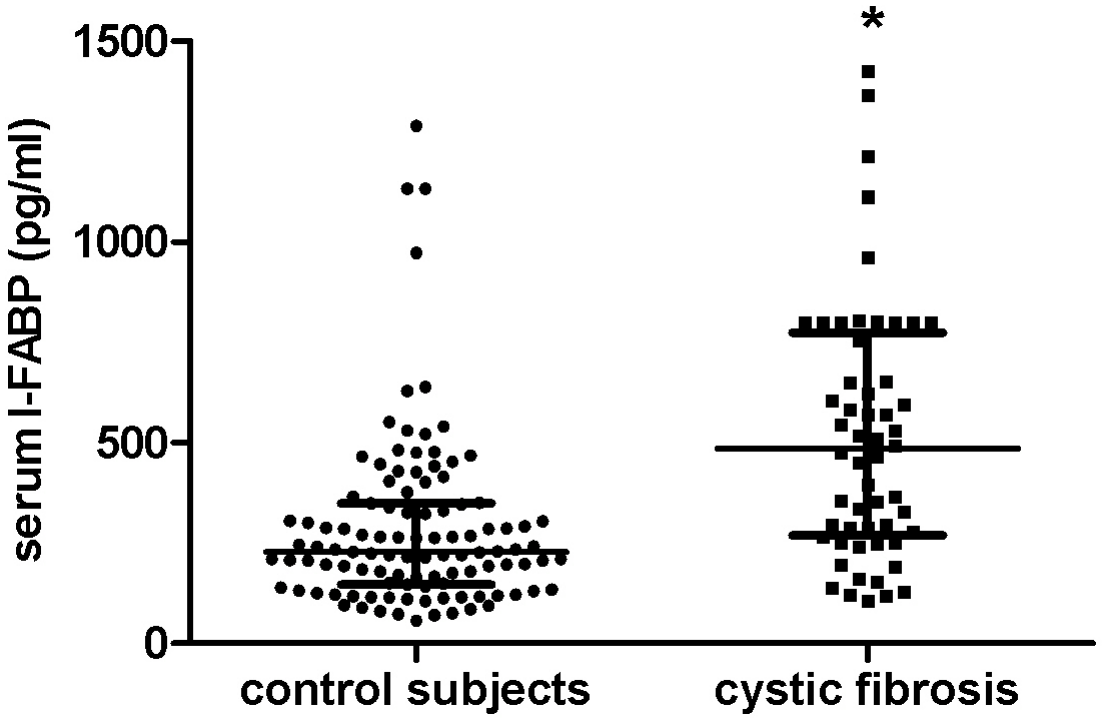
Serum I-FABP levels were significantly elevated in cystic fibrosis patients as compared with control subjects. Boxplot of serum I-FABP concentrations in the study groups, showing the median, the 25^th^ percentile, the 75^th^ percentile and the range of values. * Significant difference, p<0.001.

**Fig 2:**
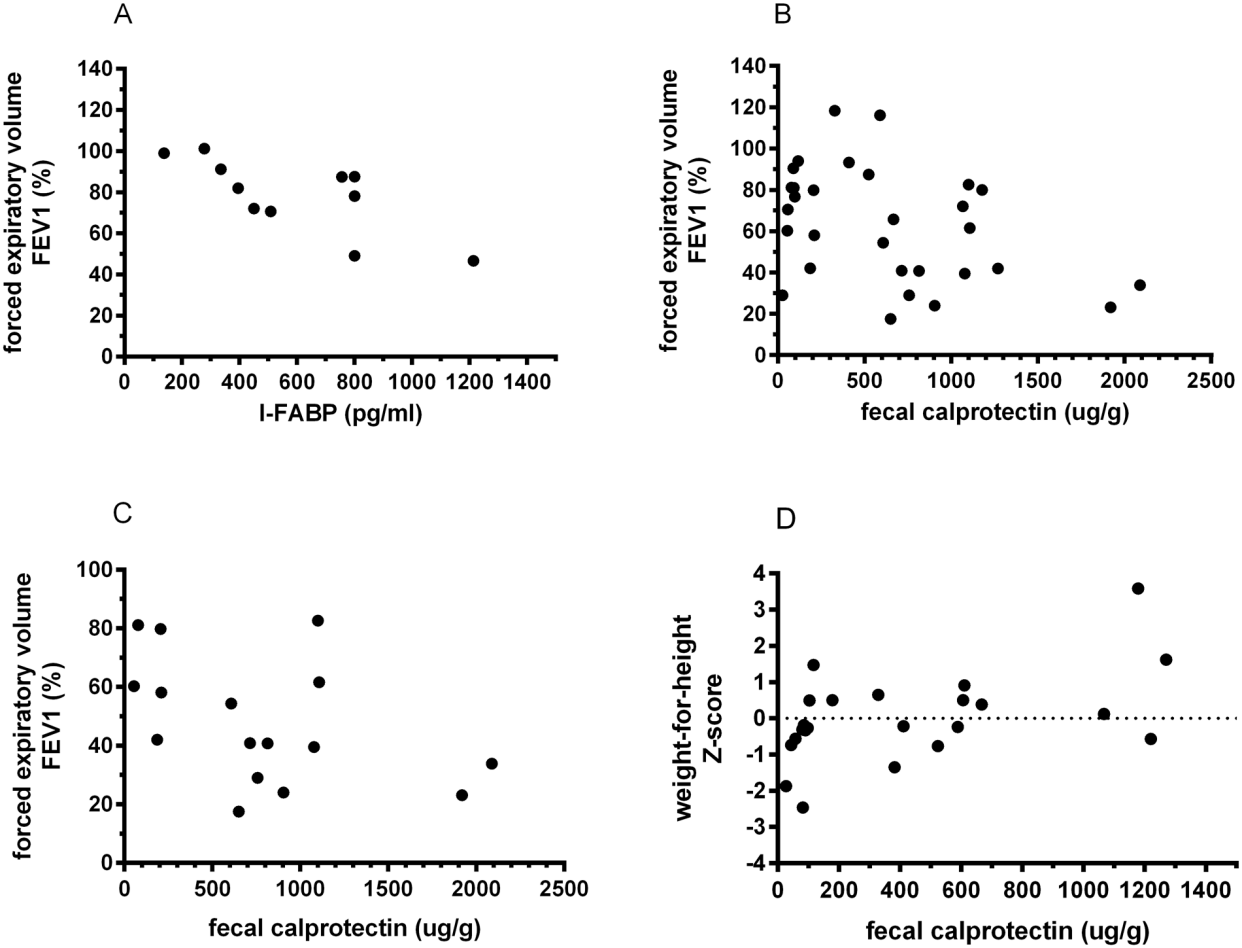
Serum I-FABP level correlated inversely with forced expiratory volume (FEV1) in children with cystic fibrosis (CF) (A). Faecal calprotectin level correlated inversely with FEV1 in the total CF group (B) and in CF adults (C). Faecal calprotectin level was significantly correlated with weight-for-height Z-score in CF children (D).

Serum I-FABP levels were significantly elevated in CF patients (486 pg/ml [270–774]) compared with control subjects (228 pg/ml [147–349], p<0.001, Fig 1). The I-FABP level was not influence by age or gender. We evaluated whether enterocyte destruction contributes to a decline in lung function. In CF children, serum I-FABP level correlated negatively with lung function (FEV1 r = -.734, p<0.05; FVC r = -.593, p = .054; FEV1/VC r = -.508, p = .110, n = 11, Fig 2A), whereas no correlation was found in adult CF patients. BMI, BMI Z-score or height-for-weight Z-score were not related with serum I-FABP or lung function tests.

Moreover, we evaluated the effect of comorbidities and medication use on I-FABP levels. Serum I-FABP levels did not differ between patients with or without CFRD, EPI, PA colonization, malnourishment or PPI use. In addition, there was no correlation between I-FABP level and lipase dosage of PERT (p = .52, n = 28). Neither CRP (p = .81, n = 36) nor faecal calprotectin levels (p = .43, n = 9), markers for inflammation, correlated with serum I-FABP level.

Furthermore, no significant differences or trends were present in the frequency of comorbidities such as EPI, CFRD, PA colonization, poor nutritional status or the use of PPI’s, lipase dose and CRP level between patients with high versus low I-FABP levels (defined as a serum I-FABP level below or above the median value of 486 pg/ml).

### Associations with faecal calprotectin

**Fig 3:**
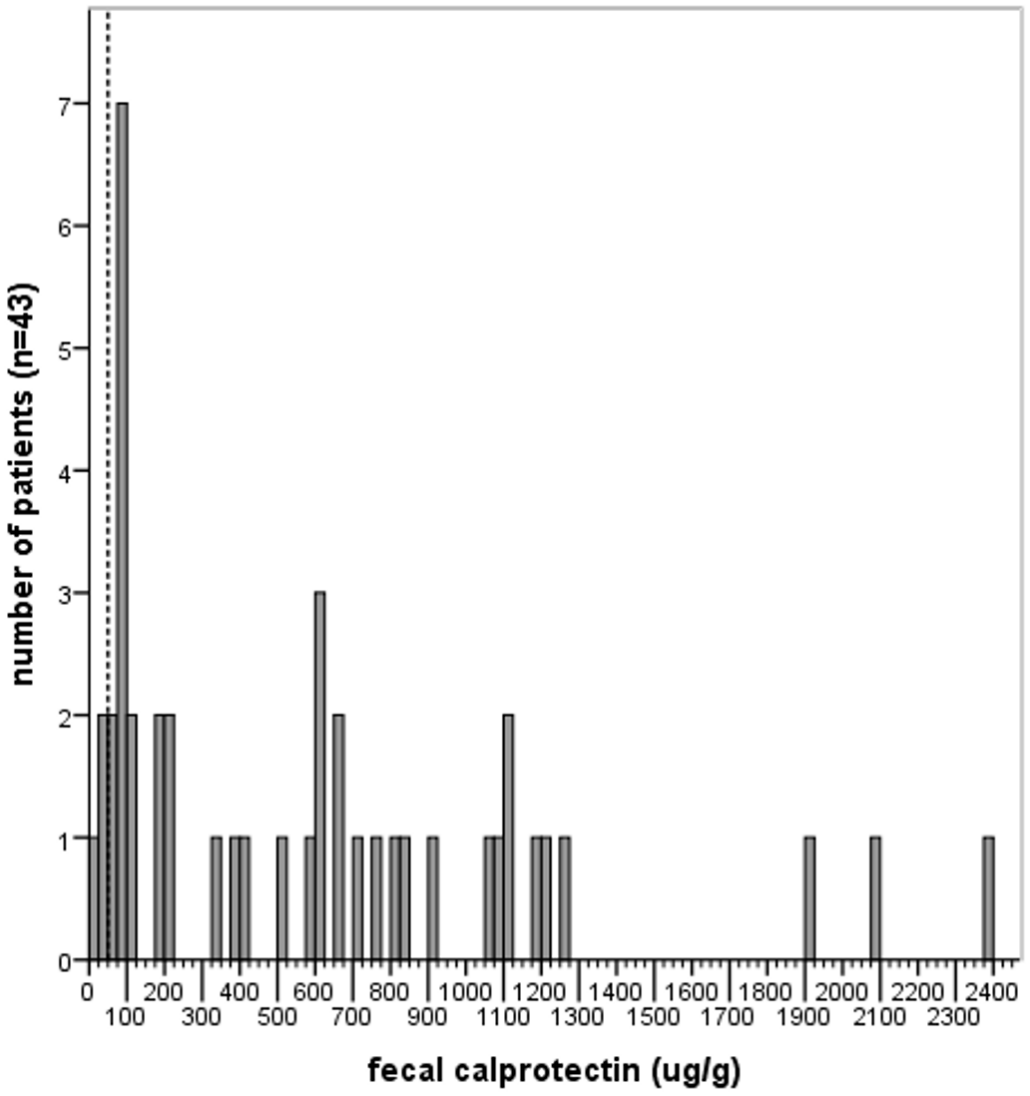
Distribution of faecal calprotectin levels in patients with cystic fibrosis (CF). The upper normal limit for faecal calprotectin (50 ug/g) is indicated by the dashed line.

Faecal calprotectin levels were elevated above the cut-off level in 40 CF patients (93%) (524 μg/g [92–905], Fig 3) Interestingly, calprotectin level correlated positively with age (r = .321, p<0.05, n = 43) while no association was found with gender (p = 0.67, n = 43). To evaluate the relation between intestinal inflammation and lung function, the correlations between faecal calprotectin level and FEV1, FVC and FEV1/VC were assessed. Calprotectin level correlated inversely with lung function in CF patients (FEV1 r = -.428, p<0.05; FVC r = -.373, p<0.05; FEV1/VC r = -.436, p<0.05, n = 33, Fig 2B), even after correction for age with multiple regression analysis. Dividing the study population into children and adults revealed a significant inverse correlation between faecal calprotectin and lung function in adult CF patients (FEV1 r = -.484, p<0.05; FVC r = -.304, p = .207; FEV1/VC r = -.509, p<0.05, n = 19, Fig 2C), while no correlation was found in children.

Since it is thought that intestinal inflammation influences nutritional status negatively, the relationship between faecal calprotectin level and nutritional status was assessed. In CF children, weight-for height Z-score was positively correlated with faecal calprotectin (r = .531, p<0.05, n = 23, Fig 2D), whereas no significant relation was found between BMI and calprotectin level in adult CF patients (r = -.346, p = .147, n = 19).

**Fig 4:**
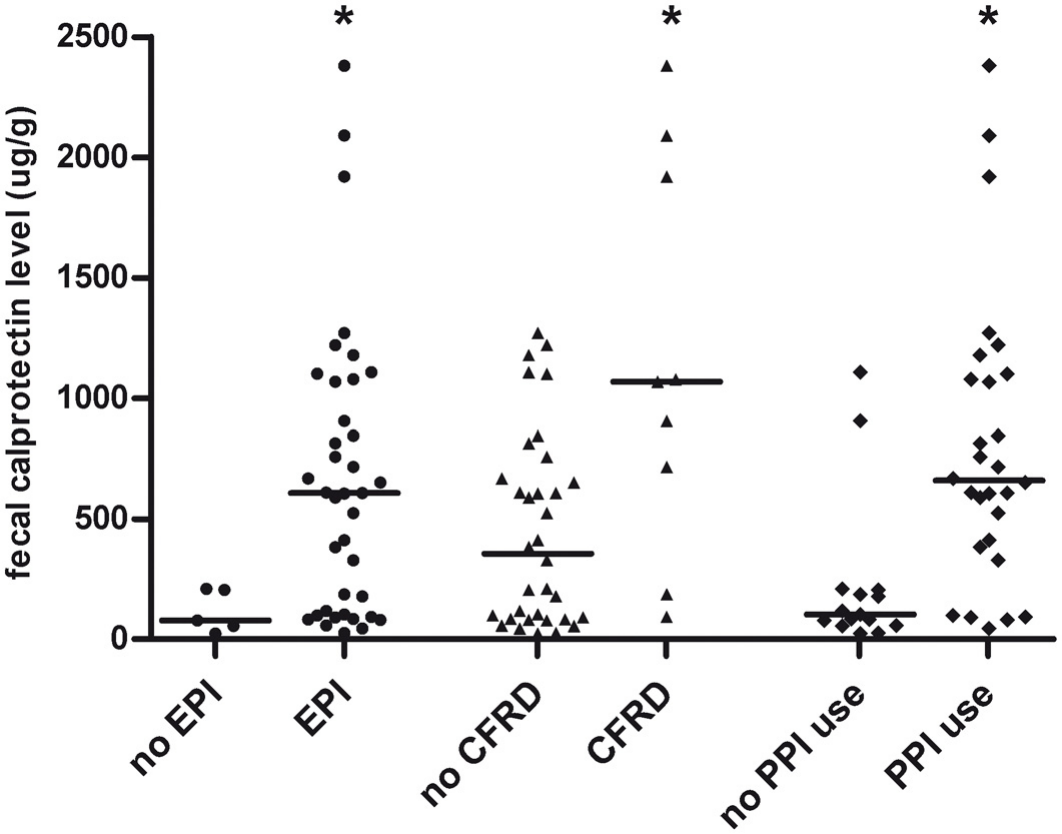
Faecal calprotectin levels were significantly elevated in cystic fibrosis (CF) patients suffering from exocrine pancreatic insufficiency (EPI) or cystic fibrosis-related diabetes (CFRD), and in CF patients using proton pump inhibitors (PPI), as compared with CF patients without these conditions.

To identify factors influencing faecal calprotectin level in CF patients, comorbidities and medication use were evaluated. Calprotectin levels were significantly elevated in EPI patients (607 μg/g [103–1070]) in comparison with exocrine pancreatic sufficient (EPS) patients (79 μg/g [40–208], p<0.05, Fig 4). Moreover, patients suffering from CFRD showed elevated calprotectin levels (1067 μg/g [451–2005]) compared with patients without CFRD (356 μg/g [84–690], p<0.05, Fig 4). Furthermore, patients using PPI’s demonstrated higher levels of faecal calprotectin (659 μg/g [389–1094]) compared with patients without PPI use (104 μg/g [58–206], p = 0.001, Fig 4). Interestingly, faecal calprotectin levels did not differ between patients with or without PA colonization (p = .55). In addition, calprotectin level did not correlate with lipase dosage of PERT (p = .12) or HbA1c (p = .49, n = 8). Linear regression analysis, taking into account age as a covariate, revealed that CFRD (B 648.5, SE 196.3, p = 0.002), PPI use (B 574.6, SE 158.2, p = 0.001) and EPI (B 862.1, SE 258.7, p = 0.002) were associated with elevated calprotectin levels in CF patients. PPI use was only present in EPI patients, consequently evident overlap was present between these patient groups.

Since it has been proposed that faecal calprotectin in CF patients might originate from the lungs as a result of swallowed sputum, we evaluated whether faecal calprotectin level correlated with calprotectin level in sputum. Median sputum calprotectin level was 27019 ug/g (8625–34185 ug/g) while median level in faeces was 524 ug/g (98–905 ug/g) in these patients (n = 23). No correlation was found between both levels. Sputum calprotectin levels correlated with age (r = -.472, p = 0.05), while no correlation was found with lung function tests when corrected for age.

## Discussion

This study provides evidence for a CF enteropathy, comprising enterocyte damage and intestinal inflammation. Interestingly, a significant inverse correlation was found between enterocyte damage and lung function in CF children, and between intestinal inflammation and lung function in CF adults. While elevated serum I-FABP levels were not related to various CF-related comorbidities or medication use, elevated faecal calprotectin levels were significantly associated with CFRD, EPI, and PPI use.

Impairment of intestinal function in CF patients is presumed due to the difficulty to achieve and maintain optimal nutritional status in these patients. Next to the role of nutritional and metabolic factors also the production of aberrant mucus has an influence on the intestinal homeostasis, especially via its influence on the microbiome. In this context, we seek evidence for the presence of enterocyte damage. As far as we know, this is the first study to analyse serum I-FABP levels in CF patients to assess intestinal epithelial damage. Serum I-FABP levels were significantly elevated in CF patients as compared with control subjects. In line with our findings, a recently developed CFTR-knockout ferret model showed villous atrophy with blunting in 50% of animals [36]. Furthermore, the brush-border enzyme acid phosphate activity has been shown to be decreased in CFTR-knockout mice compared with wild-type mice [37]. Striking is the similarity between the observed extent of enterocyte damage in CF patients in our study and that found in untreated celiac disease patients with severe villous atrophy [38, 39]. I-FABP levels were not different between patients with or without CF-related comorbidities, amongst which EPI, or medication use, being in agreement with a former study showing small intestinal injury in both EPI and EPS patients [5]. Notably, the observed enterocyte damage correlated inversely with lung function in CF children, suggesting that intestinal enteropathy and pulmonary dysfunction are connected in CF. It might be argued that lung function in adult CF patients is confounded by late complications such as, amongst others, pulmonary colonization and CFRD [40], explaining that the correlation with enterocyte damage was only found in children.

Next to enterocyte damage in the CF intestine, we evaluated the presence of intestinal inflammation. In the vast majority of CF patients (93%) faecal calprotectin level was elevated above the cut-off value, indicating intestinal inflammation. These data are supported by previous studies demonstrating elevated levels of faecal calprotectin in CF [5, 12, 13]. Interestingly, the extent of intestinal inflammation was inversely correlated with lung function in adult patients. Rumman et al evaluated this correlation in young CF children. Although lung function tests were generally worse in CF patients with high faecal calprotectin levels, there was no significant difference in line with our findings in CF children [41]. It is unclear however why this correlation was present in adult patients only.

Different potential causes for intestinal inflammation in CF can be suggested based on our study. First, the present study provides evidence for a relation between intestinal inflammation and pancreatic dysfunction, since raised levels of faecal calprotectin were associated with the presence of CFRD and EPI. In line with these findings, elevated faecal calprotectin levels have been reported in patients with EPI as compared with EPS patients [5]. The reduced bicarbonate secretion in EPI resulting in a lower pH might affect the small intestinal integrity [8], giving rise to intestinal inflammation. Furthermore, the reduced bicarbonate secretion changes the intestinal luminal fluid resulting in altered mucin secretion and mucus properties, which may contribute to intestinal inflammation. On the other hand, elevated calprotectin levels in patients with EPI might relate to the severity of the CFTR dysfunction in these patients, which may increase the risk for intestinal inflammation, independent of the EPI. Also PERT has been associated with intestinal inflammation, since an association was reported between PERT use and fibrosing colonopathy [42]. In the present study, no correlation was found between faecal calprotectin levels and lipase dosage of PERT, hence suggesting that PERT is unlikely the cause of intestinal inflammation in this CF cohort.

Another explanation for elevated calprotectin levels in CF may be the use of PPI’s. PPI use by patients without pulmonary disease was shown to be associated with increased faecal calprotectin levels [43, 44], suggesting a role for PPI’s in intestinal inflammation. Also in the current study, almost all patients with an elevated calprotectin level were using a PPI. Additionally, pancreatitis, frequently observed in CF patients [45], may contribute to the elevated calprotectin levels as high levels of faecal calprotectin have been described in non-CF chronic pancreatitis [19]. Furthermore, as calprotectin is present in sputum from CF patients [13, 46] it could be assumed that calprotectin passes the gastrointestinal tract, without conformational changes affecting its detection, contributing to elevated faecal calprotectin levels [22]. We observed that ex vivo exposure of CF sputum to an acid pH resulted in calprotectin levels half that of unexposed sputum (unpublished data), potentially supporting the assumption. However, no correlation was found between sputum and faecal calprotectin level. Moreover, calprotectin levels in faeces correlated inversely with lung function, while sputum levels did not. An acidic intestinal pH in CF patients might interfere with faecal calprotectin levels, but could only have led to an underestimation of intestinal damage in our cohort.

Another factor potentially contributing to intestinal inflammation may be bacterial overgrowth [20] and changes in the intestinal microbiome, potentially as a result of alterations in mucus production and properties. Remedies for bacterial overgrowth such as probiotics and antibiotics have been demonstrated to reduce levels of (faecal) inflammatory markers in CF [12, 47, 48], suggesting a role for bacterial overgrowth in intestinal inflammation. Interestingly, these treatments also led to increased body weight and growth in both mice and human with CF [47, 48], suggesting that adequate treatment of the CF enteropathy improves the nutritional status. Moreover, changes in the immune response resulting from the CFTR mutation may contribute to intestinal and pulmonary inflammation in CF. In this context pulmonary inflammation has been observed in CF neonates in the absence of pulmonary infection and clinically apparent respiratory symptoms [49]. A perturbed innate immune response in CF could further explain the associations between CF and celiac disease [50], Crohn’s disease [51] and severe noninfectious colitis [52].

**Fig 5:**
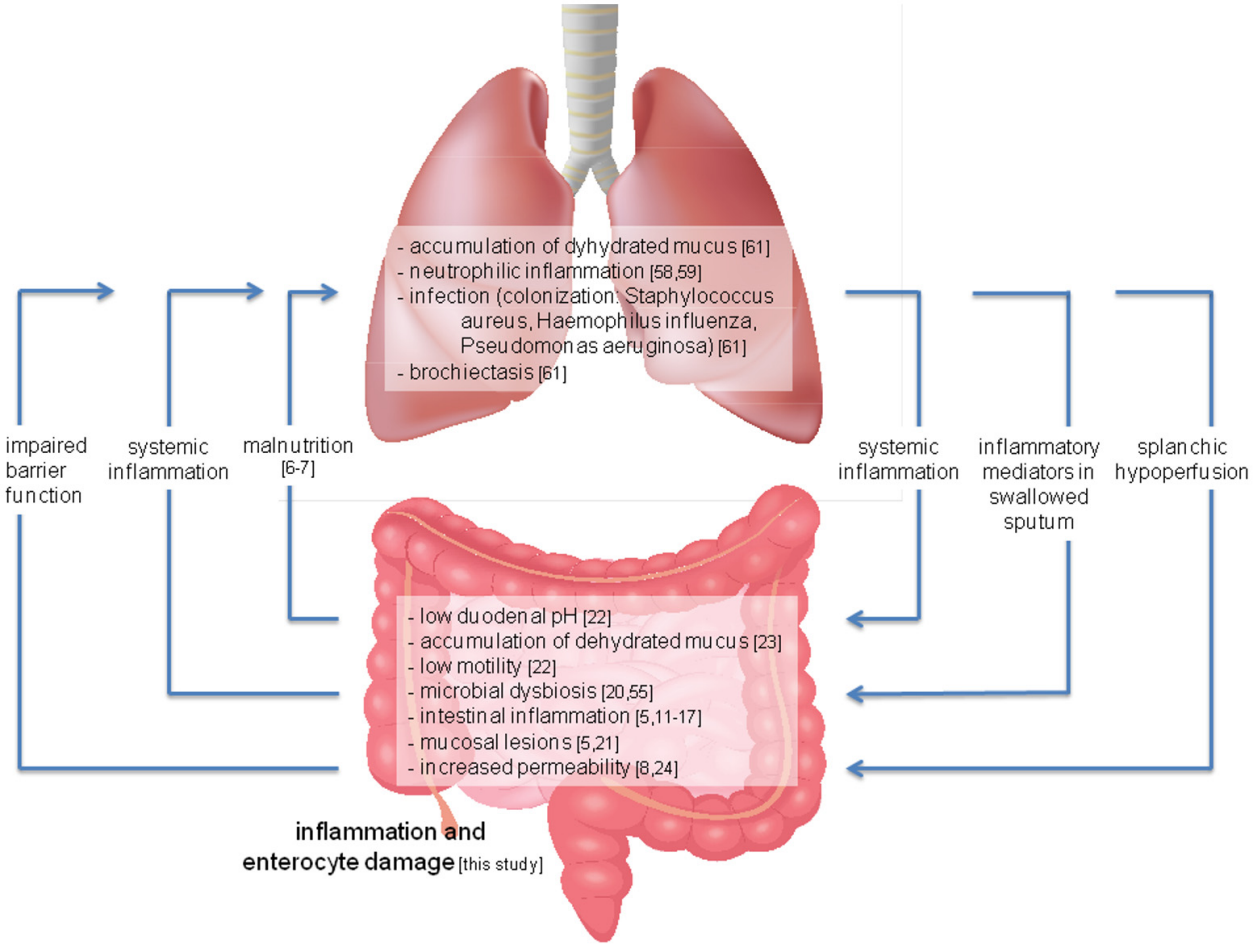
Proposed model for the interaction between intestinal and lung impairment in cystic fibrosis (CF). Intestinal alterations due to CFTR dysfunction may result in malnutrition with consistent respiratory muscle weakness and comprised innate lung defences [54], an impaired barrier function with consequent bacterial translocation to the lungs, and release of inflammatory mediators (cytokines such as interleukin (IL)-8, IL-1 and tissue necrosis factor (TNF)-a, immunoglobulins, lactoferrin, neutrophil elastase, and calprotectin [11–17]) into the systemic circulation, contributing to lung inflammation and impaired lung function. Inflammatory mediators in the lung (cytokines such as IL-8, IL-6, IL-1 and TNF-a, complement factor 5A, neutrophils, proteases (elastase), oxigen radicals, and leukotriene B4 [58, 59]) are released into the systemic circulation and will reach the intestine or, alternatively, can be swallowed with sputum (together with lung bacteria) and enter the gastrointestinal tract. Further, impaired lung function may result in splanchic hypoperfusion and intestinal hypoxia affecting the intestine. Taken together, this may contribute to intestinal inflammation and impairment and explain the complex interrelationship between CF enteropathy and lung function.

Different mechanisms can be proposed to explain the observed negative correlation between the CF enteropathy and lung function (Fig 5). A reduced intestinal barrier function, supported by reported abnormalities of tight junctions [53], resulting in bacterial translocation potentially enhances the pulmonary inflammation leading to a decline in lung function [10]. Intestinal alterations due to CFTR dysfunction may result in malnutrition with consistent respiratory muscle weakness and comprised innate lung defences [54]. This hypothesis is supported by microbiome studies in human CF infants suggesting that sequestration of microbiota by the gut results in their presence in the CF lung [9]. In line, the faecal microbiome of CFTR-knockout ferrets consists of pathogens predominantly present in the lungs [36]. Interestingly, the microbiota of the respiratory tract of CF infants contains species that require anaerobic conditions for growth, generally present in the intestine [9]. Administration of oral probiotics resulted in a reduction of pulmonary infections in CF, supporting the enteral origin of the pulmonary microbiome [47, 55]. Adequate treatment of the CF enteropathy may contribute to preservation of lung function in CF patients. Conversely, the impaired lung function in CF could be responsible for the perturbed intestinal homeostasis. In both patients with COPD and heart failure, diseases characterized by hypoxia, a compromised intestine has been reported [56, 57]. Inflammatory mediators in the lung (cytokines such as IL-8, IL-6, IL-1 and TNF-a, complement factor 5A, neutrophils, proteases (elastase), oxigen radicals, and leukotriene B4 [58, 59, 60]) may be released into the systemic circulation and reach the intestine or, alternatively, can be swallowed with sputum (together with lung bacteria) and enter the gastrointestinal tract.

Surprisingly, no correlations were found between the observed intestinal damage and nutritional status. Clinical nutritional interventions might have influenced this correlation. In particular malnourished CF patients received nutritional support correcting their weight and height impairment. This may explain the positive correlation between faecal calprotectin and weight-for-height Z-score found in the CF cohort studied.

Limitations of this study are those related to the retrospective study design; serum I-FABP and faecal calprotectin were not evaluated at the same time, and longitudinal data were not available. Furthermore, the control group consisted of patients with gastrointestinal complaints instead of a random sample of healthy subjects. However, at most this resulted in an underestimation of the difference in serum I-FABP levels between CF patients and healthy controls. In addition, it cannot be excluded that intestinal alterations and lung function correlated due to simultaneous exacerbation of CF disease; the study design does not allow concluding any causal link between intestinal alterations and lung disease. In order to gain insight in the interaction between enteropathy and lung function in CF and its chronology, serum I-FABP, faecal calprotectin, nutritional status, and lung function should be evaluated simultaneously at frequent intervals in a prospective study, taking into account pulmonary exacerbations.

In recent years, clear progress has been made in life expectancy and quality of life in CF patients. Obtaining and maintaining an optimal nutritional status and lung function remains a problem of concern in CF however. New insights in factors potentially interacting with intestinal impairment and lung function in CF provide direction for further improvement in disease control. A promising example of new developments focusing on the relationship between intestinal and pulmonary impairment are probiotics, which have been suggested to decrease the incidence of CF exacerbations and improve FEV1 [47, 55].

In conclusion, this study demonstrated enterocyte damage and intestinal inflammation in CF patients, providing further evidence for enteropathy in paediatric and adult CF patients. The observed enterocyte damage was not influenced by CF-related comorbidities or medication use, whereas intestinal inflammation was associated with EPI, CFRD, and PPI use. Interestingly, the enteropathy was inversely correlated with lung function. CF enteropathy can be proposed as a future target for therapeutic interventions in order to improve general condition and preserve lung function.

## Supporting Information

S1 File Serum I-FABP levels in CF patients. (PDF)

S2 File Faecal calprotectin levels in CF patients. (PDF)

## Abbreviations

BMI: body mass index
CF: cystic fibrosis
CFTR: cystic fibrosis transmembrane conductance regulator
CFRD: CF-related diabetes
CRP: C-reactive protein
EPI: exocrine pancreatic insufficiency
EPS: exocrine pancreatic sufficiency
FEV1: forced expiratory volume in 1 second
I-FABP: intestinal fatty acid binding protein
MUMC: Maastricht University Medical Centre
PA: Pseudomonas aeruginosa
PERT: pancreatic enzyme replacement therapy
PPI: proton pump inhibitor
