# Supplementary material for: Evidence for a Cystic Fibrosis Enteropathy
Source: PLoS One. 2015 Oct 20;10(10):e0138062. doi: 10.1371/journal.pone.0138062 (PMC4617711; doi:10.1371/journal.pone.0138062)
Supplement: S1 File — (PDF) [file pone.0138062.s001.pdf]

study number	gender	deltaF508	age	adult	I-FABP (pg/ml)	FVC	FEV1/VC	FEV1 (%)
2	1	0	45,5	1	296	80,8	56,9	47,6
3	2	-9	40,3	1	119	71,7	38,8	26,3
4	2	0	38,1	1	466	113,5	71,2	76,8
68	1	-9	39,1	1	606	56,9	51,1	28,5
5	2	0	37	1	106	65,5	87	60,3
6	1	1	36,7	1	249	57,4	72,2	42
7	2	1	35,7	1	160	57,8	75	41,3
8	2	1	34,8	1	800	69,9	81,5	54,4
9	1	0	34	1	594	78	74,7	58,4
10	1	-9	34,6	1	649	73,9	55,7	40,7
11	1	0	33,9	1	353	41,5	50,8	20,3
12	1	-9	31,1	1	357	58	40,7	23,3
72	1	-9	18,7	1	267	41	68	28
13	2	-9	25,3	1	153	83,4	62,3	50,2
14	1	-9	26,7	1	530	-9	73,7	68,8
15	1	1	23,8	1	197	94,4	81,8	74,6
16	2	1	24,3	1	366	63,6	80,6	52,2
17	2	1	23,6	1	329	44,9	58,8	25,5
19	2	1	22,2	1	251	99,8	85,9	82,9
20	2	0	24,5	1	962	89,8	97,9	86,3
21	2	0	22,7	1	288	100	90,7	89,2
22	1	0	22,2	1	518	52,4	49,8	25,9
23	2	1	22,3	1	583	42,9	62,6	26,5
24	2	0	16,8	0	800	55	88	49
25	2	1	21,5	1	1366	89,1	86,7	74,5
26	1	1	22,5	1	800	56,8	62,8	36,2
27	1	1	23,1	1	1426	104,7	85,2	88,3
28	1	0	21,5	1	806	101,4	91,5	91,1
29	1	1	19,2	1	477	75	56,02	50,4
30	2	0	21,3	1	242	35,9	60,9	23,1
32	1	1	20	1	296	55,1	92	50,7
33	2	0	19,9	1	120	98,9	96,3	92
36	1	1	19,2	1	800	83,1	68,5	55,5
69	1	0	18,3	1	802	104,6	104,3	106,3
38	1	0	15,5	0	569	-9	-9	-9
39	2	1	18	1	780	84,5	82,9	69,2
40	1	1	17,7	0	800	100,5	76,9	78,1
73	2	1	18,6	1	190	-9	-9	-9
43	1	1	13,2	0	545	-9	-9	-9
45	1	1	13,6	0	800	92,9	93,2	87,6
46	1	1	13,2	0	450	85,4	83,3	72
48	1	1	12,5	0	756	95,4	90,5	87,4
49	2	1	9,9	0	509	73	97,8	70,6
50	2	1	9,4	0	335	90,5	99,5	91,2
51	2	0	10,9	0	395	91,2	88	81,9

55	2	1	7,6	0	1214	54,8	86,6	46,6
57	2	1	9,8	0	138	102,3	93,7	99
58	1	1	9,2	0	278	95,7	103,7	101,2
60	2	0	4,9	0	571	-9	-9	-9
64	1	1	3,2	0	252	-9	-9	-9
66	1	1	0,9	0	494	-9	-9	-9
67	1	1	0,8	0	289	-9	-9	-9
74	2	1	2,5	0	129	-9	-9	-9
75	1	0	0,9	0	623	-9	-9	-9
76	2	0	0,8	0	1113	-9	-9	-9
77	2	1	0,7	0	652	-9	-9	-9

BMI adults	BMI z-score	height z-sco	weight/heigt z-sco	malnutrition	CFRD	PI	PERT
19,0	-9	-9	-9	0	0	1	1
21,5	-9	-9	-9	0	1	1	1
27,0	-9	-9	-9	0	0	0	0
20,3	-9	-9	-9	0	0	0	0
29,1	-9	-9	-9	0	0	0	0
20,5	-9	-9	-9	0	1	1	1
20,5	-9	-9	-9	0	0	1	1
25,1	-9	-9	-9	0	0	1	1
23,0	-9	-9	-9	0	0	0	0
22,6	-9	-9	-9	0	0	1	1
22,2	-9	-9	-9	0	1	1	1
17,8	-9	-9	-9	1	0	1	1
22,1	-9	-9	-9	0	0	1	1
18,9	-9	-9	-9	0	1	1	1
19,5	-9	-9	-9	0	0	1	1
19,3	-9	-9	-9	0	1	1	1
17,5	-9	-9	-9	1	0	0	0
19,3	-9	-9	-9	0	0	1	1
25,0	-9	-9	-9	0	1	1	1
23,4	-9	-9	-9	0	0	0	0
22,6	-9	-9	-9	0	0	0	0
18,4	-9	-9	-9	1	1	1	1
18,8	-9	-9	-9	0	1	1	1
16,7	-1,71	-3,42	-0,45	1	0	1	1
19,0	-9	-9	-9	0	0	1	1
22,0	-9	-9	-9	0	1	1	1
22,3	-9	-9	-9	0	0	1	1
19,3	-9	-9	-9	0	0	0	0
16,8	-9	-9	-9	1	0	1	1
19,4	-9	-9	-9	0	1	1	1
15,4	-9	-9	-9	1	1	1	1
21,2	-9	-9	-9	0	0	0	0
15,9	-9	-9	-9	1	0	1	1
20,4	-9	-9	-9	0	0	0	0
16,9	-1,06	-1,63	-0,7	0	0	1	1
20,8	-9	-9	-9	0	0	1	1
19,1	-0,58	-2,06	0,35	1	1	1	1
20,6	-9	-9	-9	0	0	1	1
23,3	3,72	-2,46	4,03	0	0	1	1
15,8	-1,31	-1,5	-0,74	1	0	1	1
16,5	-0,71	-2,16	0,12	0	1	1	1
15,0	-1,55	-2,32	-0,77	1	0	1	1
16,2	-0,98	-1,13	-0,65	0	0	1	1
15,1	-0,55	-1,73	-0,29	0	0	1	1
15,2	-0,92	-0,89	-0,72	0	0	1	1

14,3	-0,77	-3,69	-0,82	0	0	1	1
17,4	0,56	0,26	0,41	0	0	1	1
14,7	-0,63	-2,3	-0,35	0	0	1	1
17,0	1,25	-1,99	1,19	0	0	1	1
17,2	1,08	-3,13	0,4	0	0	1	1
16,8	-0,26	-3,15	0,35	0	0	1	1
16,7	-0,23	0,03	-0,19	0	0	1	1
12,1	-4,17	-0,61	-4,05	1	0	1	1
15,4	-1,43	-0,28	-1,28	1	0	1	1
16,3	-0,34	-0,67	-0,28	0	0	1	1
17,6	0,64	-0,5	0,71	0	0	1	1

Pseudomonas	PPI use	Lipase	CRP
1	1	80000	64
1	1	100000	11
1	0	-9	0
0	0	-9	66
1	0	-9	19
0	0	150000	3,5
1	1	300000	24
1	1	120000	12
0	0	-9	8,4
1	0	-9	14
1	0	-9	82
0	1	500000	16
1	1	-9	-9
0	1	500000	-9
0	0	-9	11
0	0	-9	4,9
0	0	-9	1,4
0	1	350000	9,4
1	1	-9	0
0	0	-9	16
0	0	-9	3,6
1	0	-9	16
1	1	-9	-9
1	1	-9	91
1	0	150000	3,8
0	1	300000	2,5
0	1	100000	4
1	0	-9	13
0	1	-9	4,3
1	1	225000	25
0	1	225000	1,9
0	0	-9	3,8
0	1	-9	60
0	0	-9	2,9
0	1	200000	0
0	0	-9	4,8
0	1	150000	-9
1	1	-9	-9
0	1	60000	-9
0	1	-9	-9
0	1	150000	-9
0	1	100000	-9
1	0	130000	-9
0	1	170000	-9
1	1	150000	-9

0	1	80000	0
0	0	100000	-9
0	1	180000	-9
1	0	80000	-9
0	1	80000	63
0	0	100000	-9
0	0	-9	-9
0	0	20000	2
0	1	-9	-9
0	0	-9	-9
0	0	-9	-9
