# Supplementary material for: Evidence for a Cystic Fibrosis Enteropathy
Source: PLoS One. 2015 Oct 20;10(10):e0138062. doi: 10.1371/journal.pone.0138062 (PMC4617711; doi:10.1371/journal.pone.0138062)
Supplement: S2 File — (PDF) [file pone.0138062.s002.pdf]

study number	gender	deltaF508	age	adult	fecal calprotectin	sputum calprotectin	FVC
2	1	0	46,3	1	845	46579	80,8
3	2	-9	41,1	1	2380	-9	71,7
4	2	0	39,3	1	24	-9	129,9
5	2	0	37,9	1	55	-9	65,5
6	1	1	37,3	1	187	32463	57,4
7	2	1	37,1	1	814	30662	62,8
8	1	1	35,3	1	607	7567	69,9
9	1	0	35,3	1	210	28197	84,7
12	1	-9	31,9	1	650	300000	47,3
13	2	-9	26,3	1	1079	-9	58,3
17	2	1	24,4	1	758	36000	40,7
22	1	0	22,8	1	905	38391	42,9
25	2	1	22,2	1	1108	4208	87,2
26	1	1	22	1	2090	28184	55
27	1	1	15,3	0	1220	-9	-9
28	1	0	21,5	1	79	-9	93,1
30	2	0	21,3	1	1920	24406	35,9
31	1	1	21,5	1	1100	83660	100,2
32	1	1	21,1	1	715	27795	49,5
33	2	0	20,9	1	206	26243	93,2
38	1	0	17,6	0	589	8625	106,6
40	1	1	17,3	0	92	-9	100
41	1	0	16,3	0	667	73197	74
43	1	1	15,7	0	1179	33580	79
44	2	1	14,8	0	27	-9	30
46	1	1	13,2	0	1067	8975	85,4
48	1	1	12,5	0	524	30746	95,4
49	2	1	12	0	58	-9	73,8
50	1	1	12,2	0	90	25610	90,2
51	2	0	11,5	0	98	-9	88
53	1	1	10,8	0	80	-9	-9
55	2	1	9,5	0	1270	-9	45,5
56	1	1	8,9	0	329	-9	110,7
57	2	1	8,5	0	117	1453	104,8
58	1	1	8,3	0	411	6069	87,7
60	2	0	6,7	0	104	5089	-9
62	2	1	4,2	0	606	-9	-9
63	1	1	3,9	0	382	-9	-9
64	1	1	3,7	0	44	-9	-9
65	1	0	2,8	0	610	-9	-9
66	1	1	1,8	0	179	-9	-9
67	1	1	0,7	0	85	-9	-9
74	2	1	2,5	0	82	-9	-9

FEV1/VC	FEV1	weight/height	BMI	BMI	CFRD	PI	PERT	Pseudomonas	PPI	lipase
56,9	47,6	-9	19,0	-9	0	1	1	1	1	1301
38,8	26,3	-9	21,5	-9	1	1	1	1	1	1818
64,6	79,7	-9	25,1	-9	0	0	0	1	0	-9
87	60,3	-9	29,1	-9	0	0	0	1	0	-9
72,2	42	-9	20,0	-9	1	1	1	0	0	2187
68,3	40,8	-9	20,8	-9	0	1	1	1	1	5556
81,5	54,4	-9	25,1	-9	0	1	1	1	1	1967
69,2	58,1	-9	23,0	-9	0	0	0	0	0	-9
35,6	17,5	-9	17,8	-9	0	1	1	0	1	8475
59,9	39,5	-9	19,5	-9	1	1	1	0	1	10000
64,8	29	-9	21,2	-9	0	1	1	0	1	5645
56,5	24	-9	18,4	-9	1	1	1	1	0	-9
73,2	61,6	-9	18,7	-9	0	1	1	1	0	2885
62,8	33,9	-9	22,4	-9	1	1	1	0	1	4800
-9	-9	-0,57	17,7	-0,6	0	1	1	0	1	1908
88,7	81,1	-9	19,3	-9	0	0	0	1	0	-9
60,9	23,1	-9	19,4	-9	1	1	1	1	1	4592
84,5	82,6	-9	19,1	-9	0	1	1	0	1	4333
86,3	40,9	-9	15,2	-9	1	1	1	0	1	5625
88,7	79,8	-9	21,9	-9	0	0	0	0	0	-9
107,9	116,1	-0,24	18,4	-0,9	0	1	1	0	1	3636
80	81	-9	-9,0	-9	1	1	1	0	1	-9
87,9	65,7	0,38	18,7	-0,4	0	1	1	0	1	2935
103	80	3,58	23,9	3,06	0	1	1	0	1	-9
93	29	-1,87	13,4	-2,4	0	1	1	1	0	4839
83,3	72	0,12	16,5	-0,7	1	1	1	0	1	4289
90,5	87,4	-0,77	15,0	-1,6	0	1	1	0	1	3413
96,7	70,5	-0,56	15,8	-1	0	1	1	1	0	3881
99,1	90,5	-0,33	15,6	-1,1	0	1	1	0	1	5592
82,2	76,7	-0,27	16,2	-0,5	0	1	1	1	1	4335
-9	-9	-0,31	16,4	-0,1	0	1	1	0	1	5291
85,5	41,9	1,62	17,9	0,9	0	1	1	0	1	3125
105,5	118,3	0,65	16,3	0,38	0	1	1	0	1	5618
90,8	94	1,47	19,2	1,66	0	1	1	0	0	2778
95,9	93,3	-0,22	15,0	-0,4	0	1	1	0	1	8182
-9	-9	0,49	15,9	0,44	0	1	1	1	0	3883
-9	-9	0,5	15,9	0,51	0	1	1	0	1	5202
-9	-9	-1,35	14,6	-1	0	1	1	0	1	9125
-9	-9	-0,74	15,3	-0,3	0	1	1	0	1	5926
-9	-9	0,91	17,2	0,91	0	1	1	0	1	5484
-9	-9	0,5	17,8	0,7	0	1	1	0	0	8807
-9	-9	-0,19	16,7	-0,2	0	1	1	0	0	-9
-9	-9	-2,46	13,4	-2,4	0	1	1	0	0	18519

HbA1C
-9
9,8
-9
-9
6,5
-9
-9
-9
-9
6,7
-9
7,3
-9
6,8
-9
-9
6,8
-9
9,7
-9
-9
6,85
-9
-9
-9
-9
-9
-9
-9
-9
-9
-9
-9
-9
-9
-9
-9
-9
-9
-9
-9
-9
-9
